# Supplementary material for: Force transmission by retrograde actin flow-induced dynamic molecular stretching of Talin
Source: Nat Commun. 2023 Dec 20;14:8468. doi: 10.1038/s41467-023-44018-z (PMC10733299; doi:10.1038/s41467-023-44018-z)
Supplement: Supplementary file 1 — Supplementary Information [file 41467_2023_44018_MOESM1_ESM.pdf]

Supplementary Materials

Supplemental Table 1: Summary of the motions of EGFP-xTalin1 SiMS in each XTC cell. The data from all EGFP-xTalin1 SiMS are shown in Fig. 1d.

EGFP-xTalin1 SiMS in movies acquired with 2-s interval and a 120-s time-window

	Flow (%)	Stationary (%)	Stationary to flow (%)	Flow to stationary (%)	Unclassified (%)	Retrograde flow speed (nm/s)
Cell 1 (n = 433)	37.6	49.9	6.93	2.31	3.23	31
Cell 2 (n = 232)	40.9	43.1	9.48	2.59	3.88	33
Cell 3 (n = 475)	31.8	53.5	6.53	3.79	4.42	24
Total 1140	35.9	50.0	7.28	2.98	3.86	

Supplemental Table 2: Summary of the motions of EGFP-xTalin1 (N-tag) and xTalin1-EGFP (C-tag) SiMS observed in fast tracking movies.

EGFP-tagged xTalin1 SiMS in movies acquired with 100-ms interval and a 10-s time-window

	Flow (%)	Stationary (%)	Stationary to flow (%)	Flow to stationary (%)	Back-and-forth (%)	Unclassified (%)
EGFP-xTalin1 (N-tag) (n = 155, 5 cells)	32.9	47.1	9.68	3.87	Not detectable with the N-tag probe	5.81
xTalin1-EGFP (C-tag) (n = 153, 12 cells)	37.3	39.9	*11.9	1.96	†6.54	2.61

All speckles with lifetimes ≧ 20 frames (2 sec) that existed in lamellipodia excluding the leading edge were measured. The percentages indicated by symbols, * and †, were used to calculate a rate constant k_b (see Methods).

Supplemental Table 3: Model Parameter Values

Variable	Reference Value	Description
k_{folded}	10^4 pN/μm	Folded rod domain spring constant
$l_{folded,0}$	2 nm	Folded rod domain equilibrium length
n_{talin}	145	Number of amino acids per rod domain (Yao et al., 2016)
b	0.38 nm	Size of single amino acid (Su and Purohit, 2009)
$k_{unbind,0}$	0.17 s^{-1}	Experiments in this study
Δx_{unbind}	0.514 nm	Fitted
$k_{unfold,0}$	2.5×10^{-5} s^{-1}, varied	Representative for talin rod domain (Yao et al., 2016)
Δx_{unfold}	4.1 nm, varied	Representative for talin rod domain (Yao et al., 2016)
Δt	10^{-4} s	Timestep for master equation method
Δt_{pull}	10^{-6} s	Timestep for talin pulling simulations
ζ	0.0565 pN s μm^{-1}	Friction coefficient in talin pulling simulations

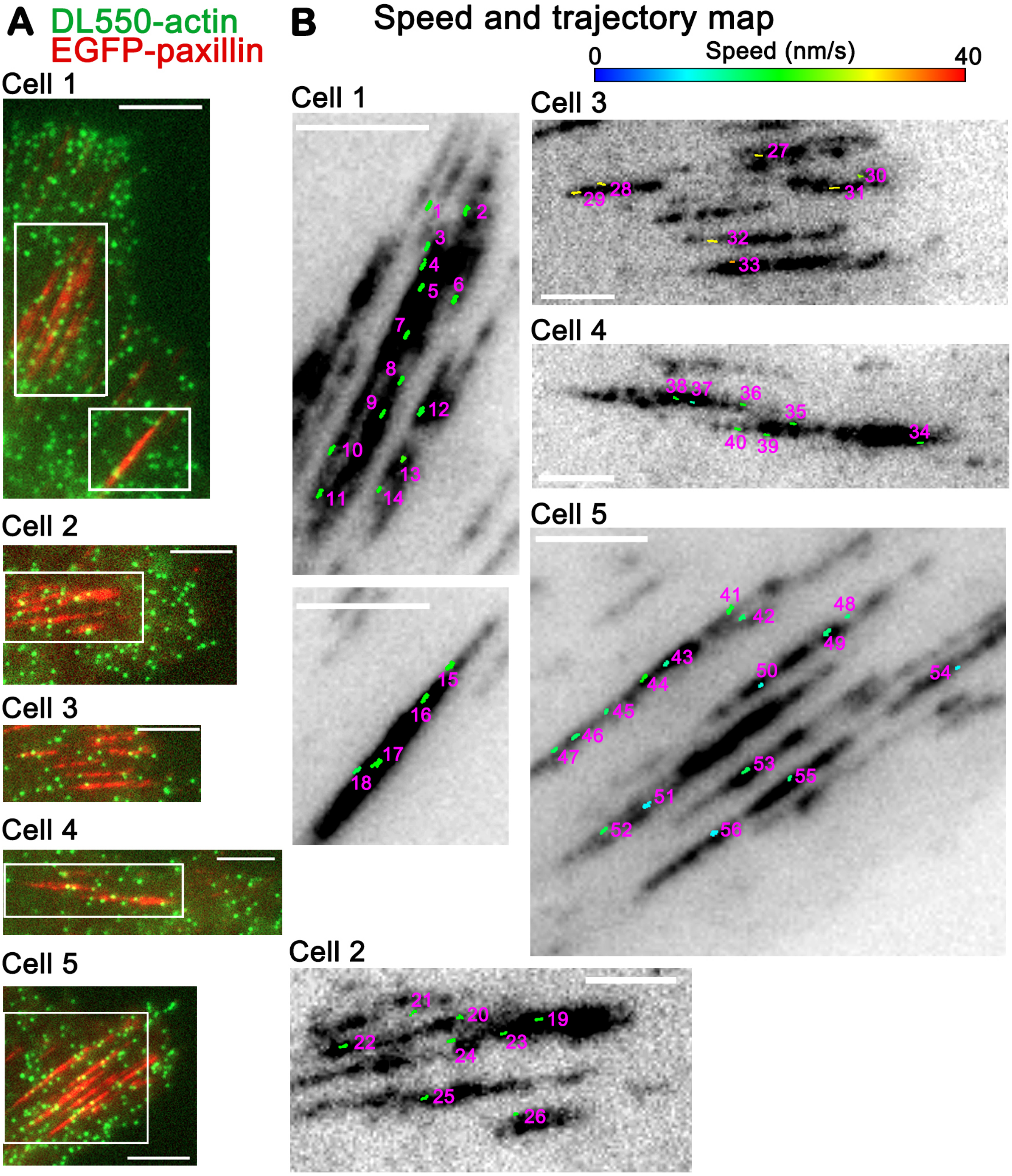
A
DL550-actin
EGFP-paxillin
Cell 1
Cell 2
Cell 3
Cell 4
Cell 5
B
Speed and trajectory map
0
Speed (nm/s)
40
Cell 1
Cell 3
Cell 4
Cell 5
Cell 2

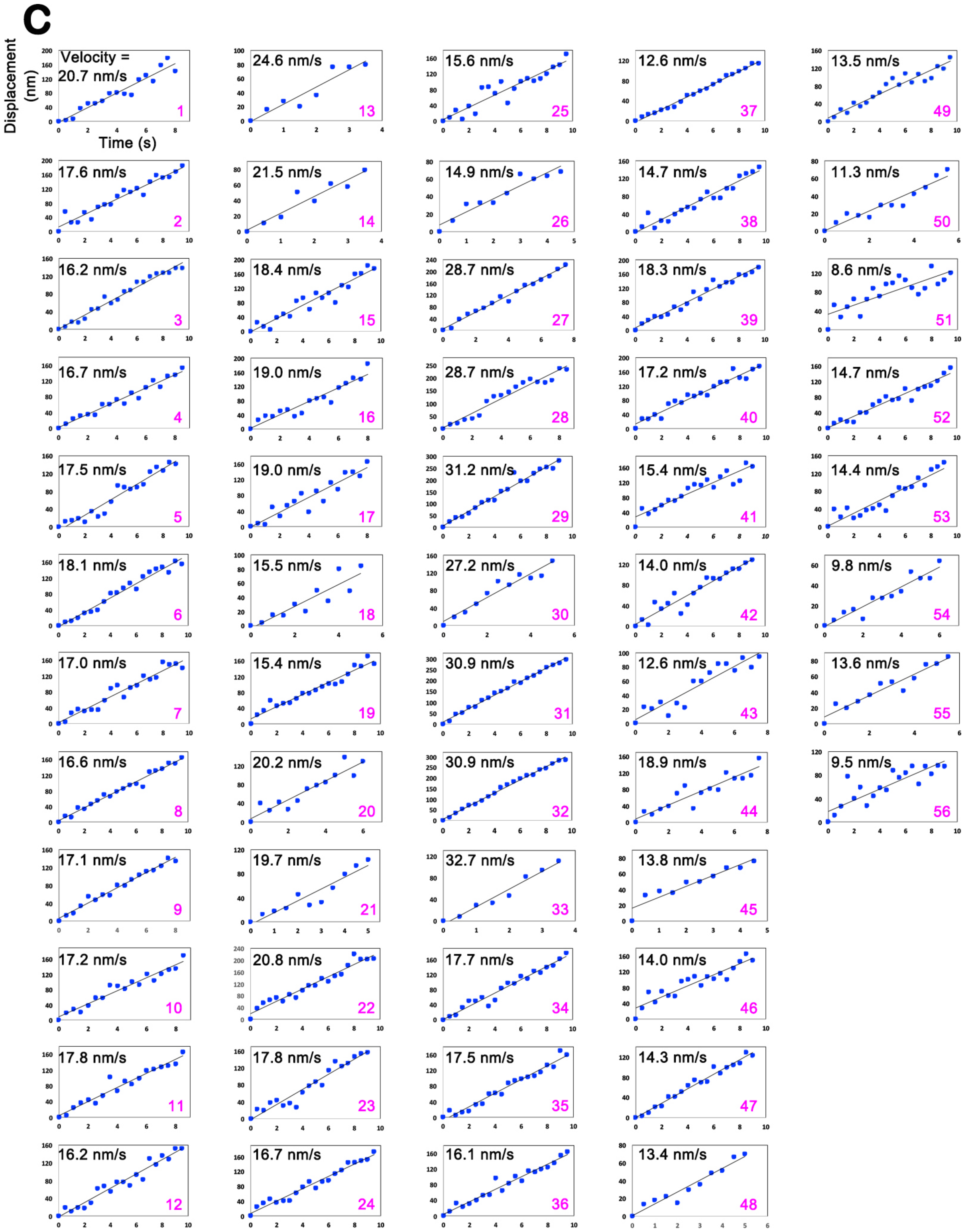
C
Displacement (nm)
Time (s)
Velocity = 20.7 nm/s
1
17.6 nm/s
2
16.2 nm/s
3
16.7 nm/s
4
17.5 nm/s
5
18.1 nm/s
6
17.0 nm/s
7
16.6 nm/s
8
17.1 nm/s
9
17.2 nm/s
10
17.8 nm/s
11
16.2 nm/s
12
24.6 nm/s
13
21.5 nm/s
14
18.4 nm/s
15
19.0 nm/s
16
19.0 nm/s
17
15.5 nm/s
18
15.4 nm/s
19
20.2 nm/s
20
19.7 nm/s
21
20.8 nm/s
22
17.8 nm/s
23
16.7 nm/s
24
15.6 nm/s
25
14.9 nm/s
26
28.7 nm/s
27
28.7 nm/s
28
31.2 nm/s
29
27.2 nm/s
30
30.9 nm/s
31
30.9 nm/s
32
32.7 nm/s
33
17.7 nm/s
34
17.5 nm/s
35
16.1 nm/s
36
12.6 nm/s
37
14.7 nm/s
38
18.3 nm/s
39
17.2 nm/s
40
15.4 nm/s
41
14.0 nm/s
42
12.6 nm/s
43
18.9 nm/s
44
13.8 nm/s
45
14.0 nm/s
46
14.3 nm/s
47
13.4 nm/s
48
13.5 nm/s
49
11.3 nm/s
50
8.6 nm/s
51
14.7 nm/s
52
14.4 nm/s
53
9.8 nm/s
54
13.6 nm/s
55
9.5 nm/s
56

Supplemental Figure 1: Actin SiMS continuously move at similar flow speeds in the center of FAs in XTC cells. **A.** Merged images of DyLight550-labeled actin (DL550-actin) SiMS (green) and EGFP-paxillin (red) that marks FAs. DL550-actin SiMS were acquired with a 500 ms exposure time for 10 sec and an unattenuated 100-W mercury illumination (20 frames). Bar＝5 μm. **B.** Speed and trajectory maps of DL550-actin SiMS in lamellipodia containing FAs. DL550-actin SiMS in the overlapping region with the signal of EGFP-paxillin were analyzed. Lines indicating the trajectories of DL550-actin SiMS observed within a 10-s time window are shown in images of EGFP-paxillin. Colors of lines indicate the speed of speckles. Subpixel localization of the centroids of DL-actin SiMS was determined with the two-dimensional Gaussian fit model of Speckle TrackerJ[16,24]. Bars＝2.5 μm. **C.** Displacement of DL550-actin SiMS with a 10-s time window (20 frames) are plotted against time. The SiMS with numbers correspond to numbered actin SiMS in **B**. The standard deviation of the difference between the measured displacement of actin SiMS and the estimated displacement by the actin flow calculated by linear approximation is ±11.7 nm, which is comparable to the low localization error of our previously reported nanometer-scale displacement measurement[16].

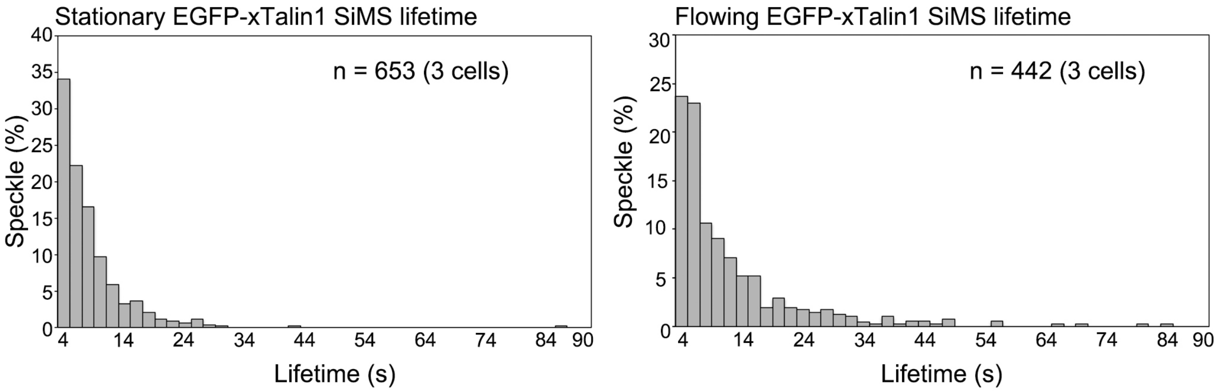

Supplemental Figure 2: The lifetime distributions of stationary xTalin1 SiMS entering a diffusing state (left) and flowing xTalin1 SiMS entering a diffusing state (right).

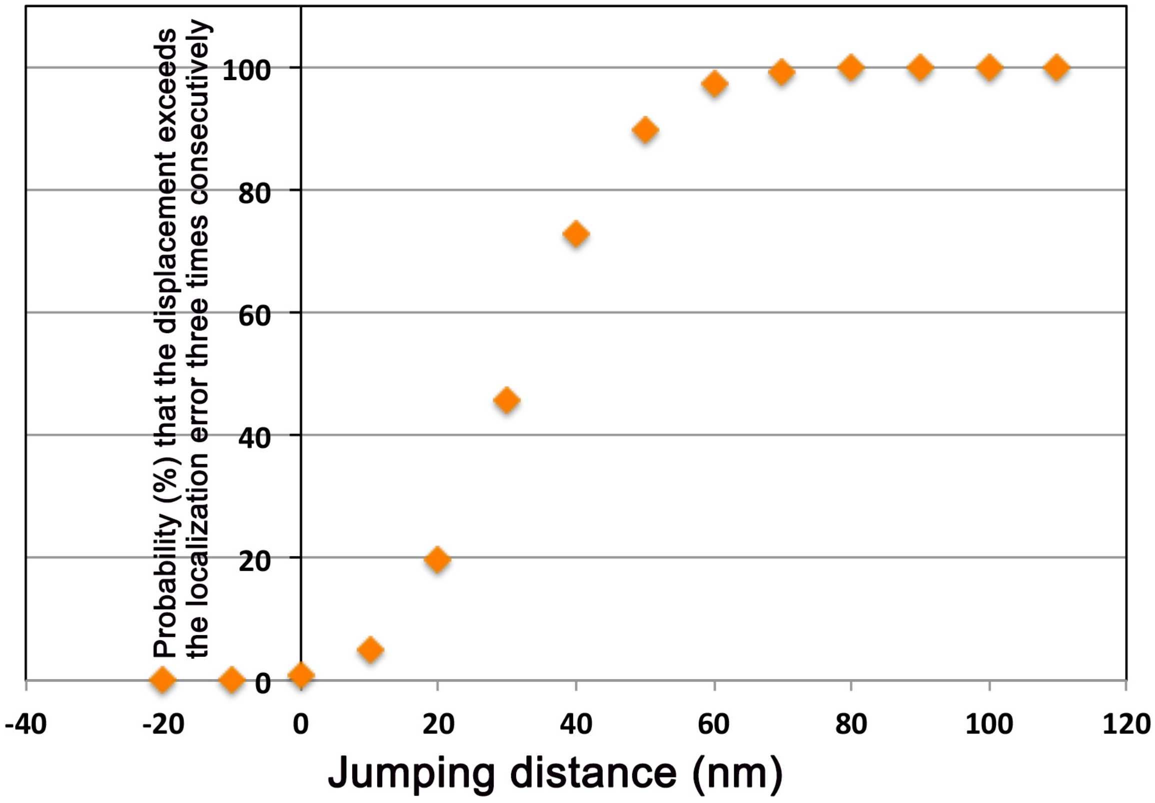

Supplemental Figure 3: The sensitivity of the jumping distance measurement shown in Fig. 2, c and d. The criteria for xTalin1 SiMS switching from stationary to flowing is that the displacement of xTalin1 SiMS in the flow direction from the stationary position exceeds the localization error of 18.6 nm three times continuously (see Methods). The graph shows the probability (%) that the displacement from the stationary position exceeds the localization error (18.6 nm) three times continuously for each jumping distance of xTalin1 flowing at 25 nm/s. The probability of the displacement exceeding the localization error was calculated using NORMDIST function in Microsoft Excel.

The Δx for the C-terminal tag on xTalin1 (xTalin1-EGFP, Fig. 2c) was 31.1 nm on average, suggesting that the C-terminal EGFP tag on xTalin1 might shift slightly in the flow direction. This is presumably due to the conformation change of Talin from a 15-nm globular to a ~60-nm open form upon F-actin binding [25]. Regarding the N-terminal tag Δx, we estimate from the previous study[25] that the conformation change of the N-terminal FERM domain upon dissociation from integrin is negligible.

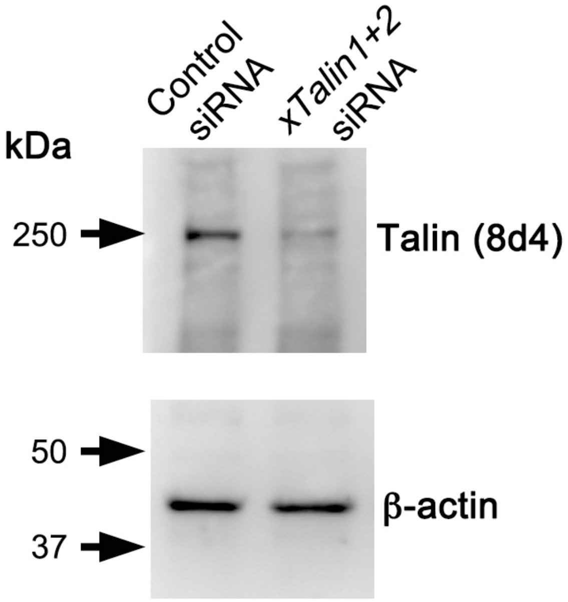

Supplemental Figure 4: Western blotting of whole cell lysates of A6 cells treated with control siRNA or *xTalin1+2* siRNA for Talin by using a mouse monoclonal anti-Talin antibody (clone 8d4). A mouse monoclonal antibody against ß-actin (clone AC-74) was used for loading control. The data shown are representative of three independent experiments.

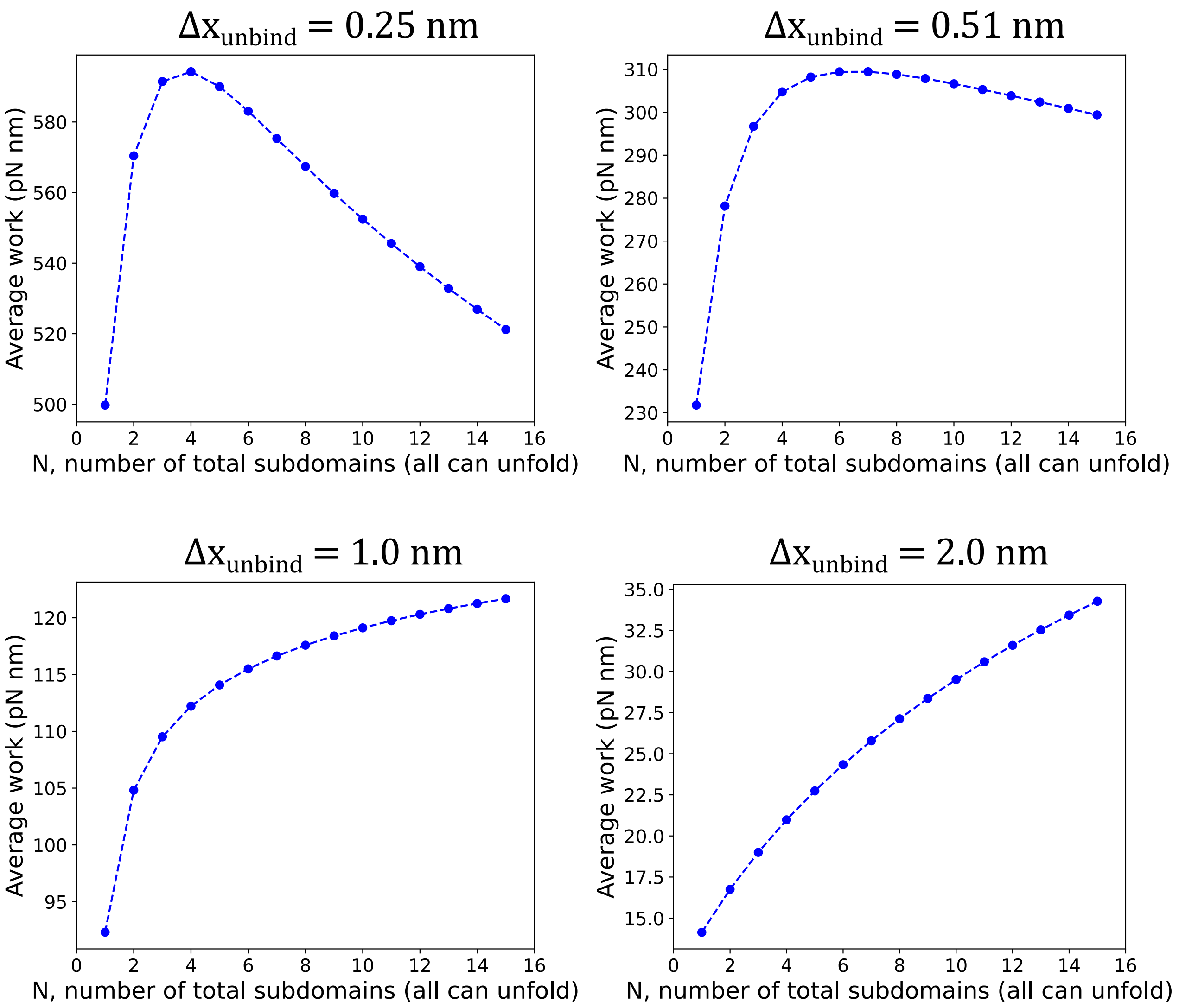

Supplemental Figure 5: Dependence of work done by Talin, as a function of integrin unbinding parameter Δx_{unbind} and length of Talin chain (number of rod subdomains, N). Simulated work calculated as in Fig. 4a by the numerical solution of the master equation of a chain of identical Talin subdomains, all assumed to be capable of unfolding. Other parameters from Table S3, retrograde flow speed 20 nm/s, and θ=45°. The smallest value of Δx_{unbind}, corresponding to the strongest Talin-integrin link, showed an optimum (maximum work) at 4 rod subdomains. This optimal number of rod subdomains increases with Δx_{unbind}. The reference Δx_{unbind} used in Fig. 4 is 0.51 nm.

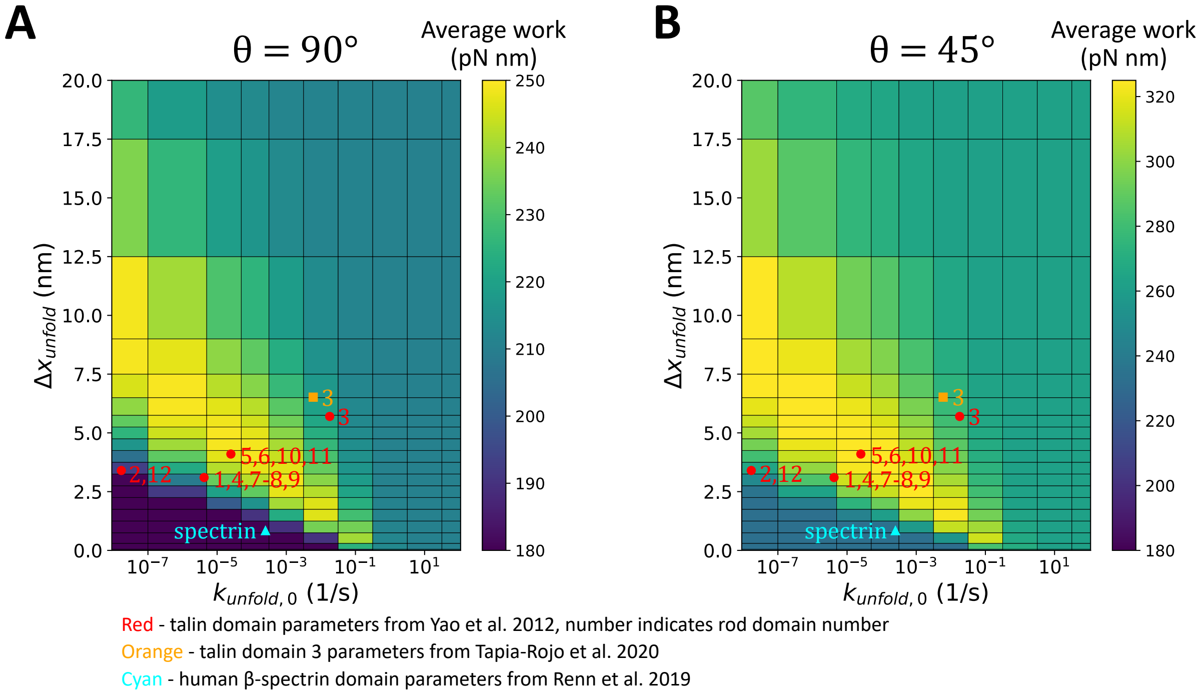

Supplemental Figure 6: Simulations of the Talin clutch show optimal Talin rod unfolding parameters. Heat map of simulated average work done by Talin on actin as shown in Fig. 4a. Results of the numerical solution of the master equation for a chain of 12 identical Talin subdomains over a range of rod subdomain unfolding parameters Δx_{unfold} and $k_{unfold,0}$ for both (A) θ=90° and (B) θ=45°. Other model parameters from Table S3 and retrograde flow speed 20 nm/s. Red circles correspond to experimentally-measured values for Δx_{unfold} and $k_{unfold,0}$ for Talin subdomains taken from Yao et al.(Yao et al., 2016), where numbers indicate the Talin rod subdomain index starting from the rod subdomain closest to the N-terminal; the orange square indicates the values for Talin rod subdomain 3 taken from Tapia-Rojo et al.(Tapia-Rojo et al., 2020); the cyan triangle indicates the values for the human β-spectrin domain taken from Renn et al.(Renn et al., 2019). Most of the experimentally-measured values Δx_{unfold} and $k_{unfold,0}$ for Talin are close to the ridge of maximum work.

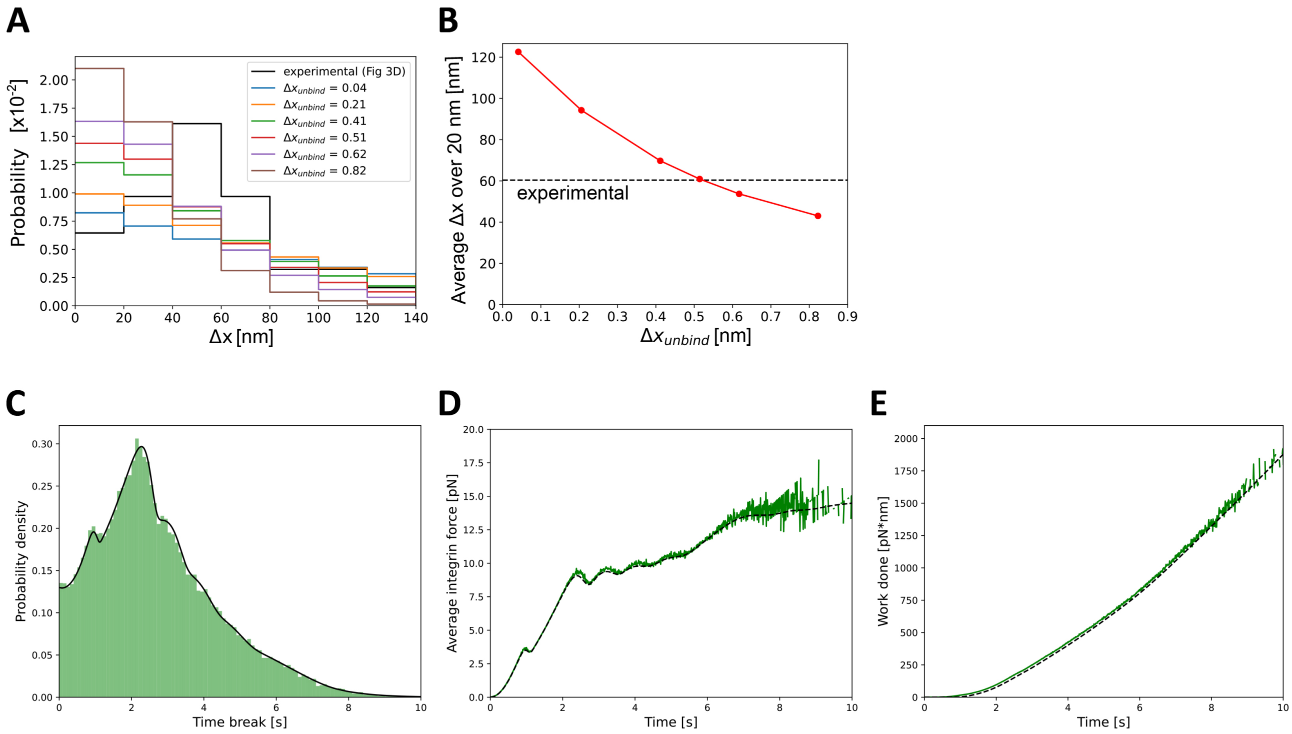

Supplemental Figure 7: Calibration of Δx_{unbind} and simulation consistency check. (A) Probability distribution of unbinding distances calculated from the master equation method as a function of Δx_{unbind} and comparison to Fig. 2D. Other parameter values as in Table S3. (B) Selection of $\Delta x_{unbind} = 0.514$ nm as the value in simulations (red sold line) that closely reproduces the experimental average unbinding distance (black dashed line), considering unbinding distances larger than 20 nm. (C-E) Agreement between results from the master equation method (black line) and from ~60,000 Talin pulling simulations (green histogram/lines) for the probability density of the breaking time (C), for the average force on the integrin bond as a function of time (D), and for the work done on actin as a function of time (E). Both types of simulations were performed on a 12-domain talin chain using unfolding parameters for each domain taken from Yao et al.(Yao et al., 2016). Increasing fluctuations with time for the Talin pulling simulations in (D) and (E) are due to low sampling in this region because of the low probability of the chain getting to this time before unbinding

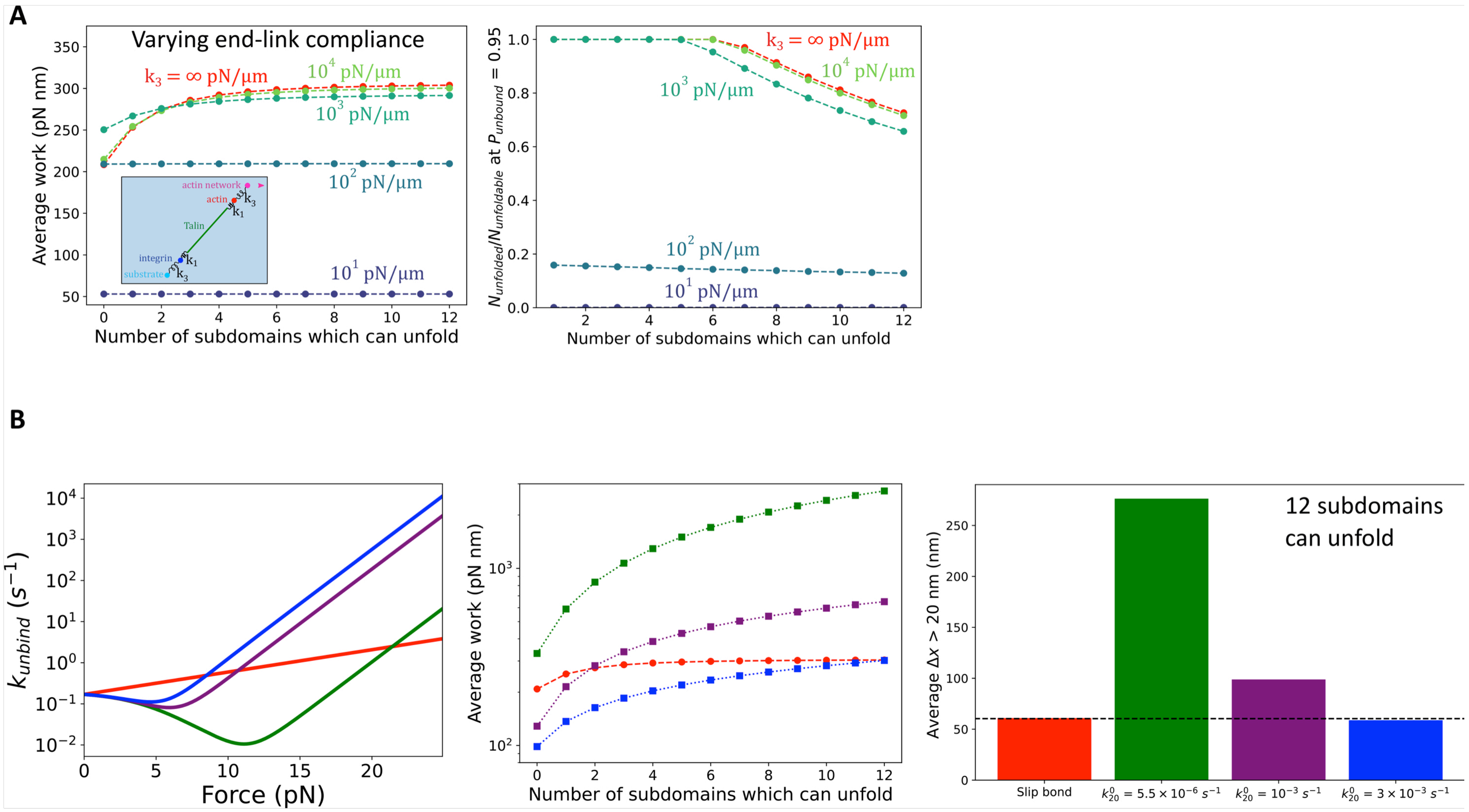
A
Varying end-link compliance
Average work (pN nm)
$k_3 = \infty$ pN/μm
10^4 pN/μm
10^3 pN/μm
10^2 pN/μm
10^1 pN/μm
actin network
actin
Talin
integrin
substrate
Number of subdomains which can unfold
$N_{unfolded}/N_{unfoldable}$ at $P_{unbound}$ = 0.95
B
k_{unbind} (s^{-1})
Force (pN)
Average work (pN nm)
Average Δx > 20 nm (nm)
12 subdomains can unfold
Slip bond
k^0_{20} = 5.5 × 10^{-6} s^{-1}
k^0_{20} = 10^{-3} s^{-1}
k^0_{20} = 3 × 10^{-3} s^{-1}

Supplemental Figure 8. Simulation results checking model robustness in response to changes of model assumptions. Other parameters as in Supplemental Table 3. (A) Average work done for different end-link compliance values. In all prior simulations, the compliance of the substrate and the actin network was infinite (red curve). Finite compliance of the actin network and substrate was simulated by adding two new spring bonds: one between the integrin bead and a newly added substrate bead; the other between the actin bead and a newly added bead meant to represent the actin network. By lowering the spring constant of these new spring bonds, the work done by Talin first flattens and then is globally reduced (left panel). However, to observe a flat work done curve, the new linkers have to be so soft that only a small fraction of the Talin subdomains unfold by the time the Talin chain unbinds (right panel). (B) Assumed Integrin-Talin unbinding rates as a function of tension along Talin for reference slip bond (red-line) and for a series of slip-catch bonds (green, purple, blue lines) whose form was implemented with a two state model (left panel). The unbinding rate at zero force was fixed at the experimentally-measured rate. Average work for all four cases shows an increase with the number of subdomains which can unfold, with catch-slip bonds exhibiting an even larger relative increase (middle panel). Average displacement of the actin bead before unbinding matches the average value from experiment for both the reference slip bond (red) and the catch-slip bond with the lowest unbinding rate minimum (blue) when all 12 subdomains can unfold (right panel). In comparison, the actin bead displacements are too large compared to experiment for curves with deeper unbinding rate minimums (purple, green).
