# Supplementary material for: Force transmission by retrograde actin flow-induced dynamic molecular stretching of Talin
Source: Nat Commun. 2023 Dec 20;14:8468. doi: 10.1038/s41467-023-44018-z (PMC10733299; doi:10.1038/s41467-023-44018-z)
Supplement: Supplementary file 3 — Description of Additional Supplementary Files [file 41467_2023_44018_MOESM3_ESM.pdf]

Description of Additional Supplementary Files

Supplementary Movie 1: Time-lapse movie of single-molecule speckles of EGFP-xTalin1 (green) in a XTC cell acquired every 2 s for 120 s. The movie is overlaid with an image of mPlum-paxillin (red) before acquisition of the SiMS imaging of EGFP-xTalin1. Scale bar, 5 µm.

Supplementary Movie 2: Time-lapse movie of dual-wavelength SiMS microscopy for EGFP-xTalin1 (green) and CF680R-actin (red) in a XTC cell acquired every 2 s for 110 s. Scale bar, 3 µm.

Supplementary Movie 3: Series of twenty discrete Talin pulling simulations, each of which end when the integrin-Talin linker dissociates and the next simulation in the sequence begins. Blue bead represents integrin, red bead represents actin, and green beads represent the endpoints of Talin subdomains. Green bonds represent folded, spring bonds while red bonds represent unfolded, freely jointed chain bonds. $\theta = 90°$ and the red, actin bead moves with speed $v = 20\ nm/s$ in the positive x-direction. Other parameters are as listed in Supplemental Table 3 with each Talin subdomain being identical and having the reference unfolding parameters (Simulation Methods section). The framerate is 24 frames per second with 0.1 s of simulation time between frames.
