# Supplementary material for: Force transmission by retrograde actin flow-induced dynamic molecular stretching of Talin
Source: Nat Commun. 2023 Dec 20;14:8468. doi: 10.1038/s41467-023-44018-z (PMC10733299; doi:10.1038/s41467-023-44018-z)
Supplement: Supplementary file 7 — Reporting Summary [file 41467_2023_44018_MOESM7_ESM.pdf]

nature portfolio

Corresponding author(s): Sawako Yamashiro

Last updated by author(s): Nov 23, 2023

Reporting Summary

Nature Portfolio wishes to improve the reproducibility of the work that we publish. This form provides structure for consistency and transparency in reporting. For further information on Nature Portfolio policies, see our Editorial Policies and the Editorial Policy Checklist.

Statistics

For all statistical analyses, confirm that the following items are present in the figure legend, table legend, main text, or Methods section.

n/a	Confirmed	
☐	☒	The exact sample size (n) for each experimental group/condition, given as a discrete number and unit of measurement
☐	☒	A statement on whether measurements were taken from distinct samples or whether the same sample was measured repeatedly
☐	☒	The statistical test(s) used AND whether they are one- or two-sided<br>*Only common tests should be described solely by name; describe more complex techniques in the Methods section.*
☒	☐	A description of all covariates tested
☐	☒	A description of any assumptions or corrections, such as tests of normality and adjustment for multiple comparisons
☐	☒	A full description of the statistical parameters including central tendency (e.g. means) or other basic estimates (e.g. regression coefficient) AND variation (e.g. standard deviation) or associated estimates of uncertainty (e.g. confidence intervals)
☐	☒	For null hypothesis testing, the test statistic (e.g. F, t, r) with confidence intervals, effect sizes, degrees of freedom and P value noted<br>*Give P values as exact values whenever suitable.*
☒	☐	For Bayesian analysis, information on the choice of priors and Markov chain Monte Carlo settings
☒	☐	For hierarchical and complex designs, identification of the appropriate level for tests and full reporting of outcomes
☐	☒	Estimates of effect sizes (e.g. Cohen's d, Pearson's r), indicating how they were calculated

Our web collection on statistics for biologists contains articles on many of the points above.

Software and code

Policy information about availability of computer code

Data collection	Image acquisitions were steered by Metamorph software (Molecular Devices) as detailed in method section.
Data analysis	Tracking of single-molecule speckles was performed using Speckle TrackerJ plug-in of ImageJ as described previously (Smith et al., Biophys J, 2011; Yamashiro et al., MBoC, 2014; Yamashiro et al., Method Cell Biol, 2015). The fluorescent intensity of EGFP was measured using Metamorph software. Microsoft Excel (2011 & 2016) and GraphPad Prism (6.07) were used for data analysis. The code for simulations is available at https://github.com/davidmrutkowski/TalinStretching.

For manuscripts utilizing custom algorithms or software that are central to the research but not yet described in published literature, software must be made available to editors and reviewers. We strongly encourage code deposition in a community repository (e.g. GitHub). See the Nature Portfolio guidelines for submitting code & software for further information.

Data

Policy information about availability of data

All manuscripts must include a data availability statement. This statement should provide the following information, where applicable:

- Accession codes, unique identifiers, or web links for publicly available datasets
- A description of any restrictions on data availability
- For clinical datasets or third party data, please ensure that the statement adheres to our policy

All the data supporting this study are available within the article and its Supplementary Information file. Source data are provided with this paper.

Research involving human participants, their data, or biological material

Policy information about studies with human participants or human data. See also policy information about sex, gender (identity/presentation), and sexual orientation and race, ethnicity and racism.

Reporting on sex and gender	Not Applicable.
Reporting on race, ethnicity, or other socially relevant groupings	Not Applicable.
Population characteristics	Not Applicable.
Recruitment	Not Applicable.
Ethics oversight	Not Applicable.

Note that full information on the approval of the study protocol must also be provided in the manuscript.

Field-specific reporting

Please select the one below that is the best fit for your research. If you are not sure, read the appropriate sections before making your selection.

☒ Life sciences ☐ Behavioural & social sciences ☐ Ecological, evolutionary & environmental sciences

For a reference copy of the document with all sections, see nature.com/documents/nr-reporting-summary-flat.pdf

Life sciences study design

All studies must disclose on these points even when the disclosure is negative.

Sample size	No statistical calculations were used to predict the sample size. We have shown the sample size information in Fig. 1C, Supplemental Tables 1 and 2, Supplemental Fig. 1 and the figure legends. Sample sizes of xTalin1 SiMS analysis (Fig. 1, Fig. 2, Supplemental Tables 1 and 2, Supplemental Fig. 1 and 2) were determined to be adequate based on extensive experience with similar experiments in our laboratory (Watanabe and Mitchison, Science, 2002; Miyoshi et al., J Cell Biol 2006; Higashida et al, Nat Cell Biol, 2013; Yamashiro et al., MBoC, 2014; Mizuno et al., PNAS, 2018).
Data exclusions	No data were excluded from the study.
Replication	We performed at least 3 independent experiments for all results and ensured that the results were reproducible.
Randomization	No randomization was performed, since all analyzed cells were selected from homogenous and linear strain.
Blinding	To collect data shown in Fig. 1, Fig. 2c and d, Fig. 3D and Fig, S2, all SiMS were used for the analysis. To collect data shown in Fig. S1B and C, all SiMS in the overlapping region with the EGFP-paxillin signal were used for the analysis. To collect data shown in Fig. 3b, e and f, all cells were used for the analysis. Therefore, no blinding was required.

Reporting for specific materials, systems and methods

We require information from authors about some types of materials, experimental systems and methods used in many studies. Here, indicate whether each material, system or method listed is relevant to your study. If you are not sure if a list item applies to your research, read the appropriate section before selecting a response.

Materials & experimental systems

n/a	Involved in the study
☐	☒ Antibodies
☐	☒ Eukaryotic cell lines
☒	☐ Palaeontology and archaeology
☒	☐ Animals and other organisms
☒	☐ Clinical data
☒	☐ Dual use research of concern
☒	☐ Plants

Methods

n/a	Involved in the study
☒	☐ ChIP-seq
☒	☐ Flow cytometry
☒	☐ MRI-based neuroimaging

Antibodies

Antibodies used

1. Mouse monoclonal anti-Talin antibody (clone 8d4, Sigma-Aldrich, T3287, Western blot 1:100)
2. Mouse monoclonal-anti-beta-actin antibody (clone AC-74, Sigma-Aldrich, A2228,Western blot 1:1000)
3. Mouse TrueBlot ULTRA: Anti-Mouse Ig HRP (Cat# 18-8817-31, eBioscience, Western blot 1:1000)

Validation

All antibodies used are standard in the field and are cited in the manuscript where relevant:
1. Use of antibody for Western blot was validated by Sigma-Aldrich using chicken gizzard extract at a dilution of 1:100.
2. Use of antibody for Western blot was validated by Sigma-Aldrich using total cell extracts of human or chicken fibroblasts at a dilution of 1:2000.
3. Use of antibody for Western blot was validated by Rockland using extracts of cancer cell lines at a dilution of 1:1000.

Eukaryotic cell lines

Policy information about cell lines and Sex and Gender in Research

Cell line source(s)

Xenopus laevis XTC cell line was previously described in Watanabe and Mitchison, Science. Xenopus laevis A6 kidney epithelial cell line were received from the laboratory of Dr. Yuko Mimori-Kiyosue (RIKEN) and was previously described in Mimori-Kiyosue et al., Genes to Cells, 2007.

Authentication

None of the cell lines used were authenticated.

Mycoplasma contamination

No mycoplasma contamination was detected.

Commonly misidentified lines
(See ICLAC register)

The cell line used in this study is not a misidentified line.
